# The DEAD/DEAH Box Helicase, DDX11, Is Essential for the Survival of Advanced Clear Cell Renal Cell Carcinoma and Is a Determinant of PARP Inhibitor Sensitivity

**DOI:** 10.3390/cancers13112574

**Published:** 2021-05-24

**Authors:** Jee Soo Park, Myung Eun Lee, Won Sik Jang, Koon Ho Rha, Seung Hwan Lee, Jongsoo Lee, Won Sik Ham

**Affiliations:** 1 Department of Urology and Urological Science Institute, Yonsei University College of Medicine, Seoul 03722, Korea; jsparkysmed@gmail.com (J.S.P.); lme0228@yuhs.ac (M.E.L.); sindakjang@yuhs.ac (W.S.J.); khrha@yuhs.ac (K.H.R.); leeseh@yuhs.ac (S.H.L.); js1129@yuhs.ac (J.L.); 2 Department of Urology, Sorokdo National Hospital, Goheung 59562, Korea

**Keywords:** renal cell carcinoma, DDX11, PARP inhibitor, olaparib, sunitinib, 786-O

## Abstract

**Simple Summary:**

*DDX11*, a helicase involved in sister chromatid cohesion, was identified as a significant biomarker of aggressive renal cell carcinoma (RCC) in our previous studies. In this study, we evaluated the molecular pathways through which DDX11 is involved in RCC cell survival. Furthermore, we assessed the sensitivity of poly (ADP-ribose) polymerase (PARP) inhibitors, which have not been used in RCC treatment, in association with DDX11 expression. *DDX11*-deficient RCC inhibited RCC proliferation, caused defects in segregation, and increased apoptosis. *DDX11*-deficient RCC was associated with increased sensitivity to PARP inhibition. *DDX11* could be a novel therapeutic and prognostic biomarker for RCC patients, and this study is the first to suggest the use of PARP inhibitors in *DDX11*-deficient RCC patients.

**Abstract:**

Genes associated with the DEAD-box helicase *DDX11* are significant biomarkers of aggressive renal cell carcinoma (RCC), but their molecular function is poorly understood. We analyzed the molecular pathways through which DDX11 is involved in RCC cell survival and poly (ADP-ribose) polymerase (PARP) inhibitor sensitivity. Immunohistochemistry and immunoblotting determined DDX11 expression in normal kidney tissues, benign renal tumors, and RCC tissues and cell lines. Quantitative polymerase chain reaction validated the downregulation of DDX11 in response to transfection with *DDX11*-specific small interfering RNA. Proliferation analysis and apoptosis assays were performed to determine the impact of *DDX11* knockdown on RCC cells, and the relevant effects of sunitinib, olaparib, and sunitinib plus olaparib were evaluated. DDX11 was upregulated in high-grade, advanced RCC compared to low-grade, localized RCC, and DDX11 was not expressed in normal kidney tissues or benign renal tumors. *DDX11* knockdown resulted in the inhibition of RCC cell proliferation, segregation defects, and rapid apoptosis. *DDX11*-deficient RCC cells exhibited significantly increased sensitivity to olaparib compared to sunitinib alone or sunitinib plus olaparib combination treatments. Moreover, *DDX11* could determine PARP inhibitor sensitivity in RCC. *DDX11* could serve as a novel therapeutic biomarker for RCC patients who are refractory to conventional targeted therapies and immunotherapies.

## 1. Introduction

The understanding of the biology of renal cell carcinoma (RCC), especially the perturbed pathways that lead to the development and growth of tumors and their multiple subtypes with distinct molecular abnormalities, has been steadily improving [1,2]. Some RCC patients (30%) present with metastatic disease at the time of initial diagnosis, and almost 30% of the patients with localized RCC develop recurrent disease during follow-up, which requires systemic therapy [3]. Treatments for advanced RCC have become markedly advanced over the past 30 years due to significant improvements in our understanding of this cancer [2]. Multiple therapeutic agents with varying mechanisms are available, and combinatorial therapy using agents with different mechanisms is now being explored. Therefore, it is crucial to provide patients with the most effective therapy and promote their quality of life [2,4]. Appropriate therapeutic effects of a combinatorial regimen along with dosage optimization will need to be ascertained to avoid treatment discontinuation due to intolerable toxicity and ensure that a marked therapeutic outcome can be achieved [5].

Although our understanding of the genomic characterization of RCC has dramatically improved, molecular profiling of RCC has not been conducted in routine clinical care, especially for selecting targeted therapies for advanced RCC [6]. None of the clinically available biomarkers can delineate advanced RCC patients who stand to benefit from specific molecule-targeted agents and individualized clinical management strategies [6,7].

In line with our long-term effort to identify genes associated with the aggressiveness of clear cell RCC (ccRCC) [8,9,10], we recently profiled archived formalin-fixed paraffin-embedded tissue samples of clinical T1 stage ccRCC [9]. The data from the total RNA sequencing of 24 ccRCC patients (12 patients with, and 12 patients without, aggressive characteristics) revealed 10 genes with the highest upregulation or downregulation in aggressive ccRCC. Among these 10 genes, *DDX11* was significantly upregulated in aggressive ccRCC and was associated with low cancer-specific survival and high recurrence rates [9]. Moreover, a prospective validation study (NCT03694912) that employed quantitative reverse transcription polymerase chain reaction (qRT-PCR) for investigating the gene expression profile of frozen tissues revealed *DDX11* to be a significant predictor of ccRCC aggressiveness [10].

*DDX11*, a member of the DEAD (Asp-Glu-Ala-Asp)/DEAH (Asp-Glu-Ala-His) box family of helicases comprising more than 40 members, is essential for the cohesion of chromosome arms and centromeres [11]. When *DDX11* is depleted, mitotic failure occurs due to replicated chromosomes failing to segregate after prometaphase arrest [11]. *DDX11* has been shown to be associated with melanomas and lung cancer [12,13]; however, no studies have been performed regarding its association with ccRCC. Furthermore, because the use of poly (ADP-ribose) polymerase (PARP) inhibitors in RCC is not clinically indicated [2], it would be helpful to expand the usage of PARP inhibitors in certain RCC patients in whom the benefits would be promising.

Thus, we aimed to elucidate the molecular pathways through which DDX11 is involved in RCC cell survival and PARP inhibitor sensitivity in RCC.

## 2. Methods

### 2.1. RCC Cell Lines and Tissues

The human RCC cell lines 786-O (cat# CRL-1932), Caki-1 (cat# HTB-46), A-498 (cat# HTB-44), and ACHN (cat# CRL-1611), and the human proximal tubular cell line, HK-2, representing normal kidney cells (cat# CRL-2190), were purchased from the American Type Culture Collection (Manassas, VA, USA). These cells were cultured in Roswell Park Memorial Institute (RPMI) or Eagle’s Minimum Essential Medium (EMEM) medium supplemented with 10% fetal bovine serum (Gibco; Thermo Fisher Scientific, Waltham, MA, USA) and 1% penicillin-streptomycin (Sigma-Aldrich, St. Louis, MO, USA) at 37 °C in a humidified atmosphere containing 5% CO_2_.

Standard immunohistochemistry analysis to assess the expression of DDX11 was performed using a mouse monoclonal antibody against human DDX11 (Sigma-Aldrich) in 6 cryopreserved human tissue samples from nephrectomy specimens (where none of the patients received preoperative chemotherapy or radiation prior to surgical excision). The samples represented normal kidney tissues, benign renal tumors (angiomyolipoma and oncocytoma), localized low-grade ccRCC, localized high-grade ccRCC, and advanced high-grade ccRCC. All DDX11 antibody-probed tissue sections were counterstained with hematoxylin.

We obtained ethical approval from the Institutional Review Board of the Yonsei University Health System (project no. 4-2013-0742) before procuring the tissue. A senior urologic pathologist with extensive experience in renal pathology (N.H.C.) evaluated the staining. Grading was based on the grading system recommended by the World Health Organization (WHO)/International Society of Urological Pathology (ISUP) [14]. Low-grade ccRCC constitutes Fuhrman or WHO/ISUP grades I and II, whereas high-grade ccRCC constitutes grades III and IV.

### 2.2. Immunoblotting and Immunofluorescence

Cells were lysed in radioimmunoprecipitation assay lysis buffer (Cell Signaling Technology, Beverly, MA, USA) to prepare total protein, and equal amounts of the protein lysate (30 µg/sample) were separated on sodium dodecyl sulfate-polyacrylamide gels and transferred onto a nylon membrane. The membranes were then probed with primary antibodies against human DDX11 (MyBioSource, San Diego, CA, USA) or β-actin (Santa Cruz Biotechnology, Dallas, TX, USA), followed by incubation with horseradish peroxidase-conjugated secondary antibody (Cell Signaling Technology). Protein bands were visualized using the Amersham ECL Prime Western Blotting Detection System (GE Healthcare, Amersham, UK). For immunofluorescence staining, cells were plated on Lab-Tek chamber slides (NUNC, Naperville, IL, USA) and received the desired treatment. At the end of the treatment, RCC cells were fixed with 4% paraformaldehyde and incubated with 0.5% Triton X-100 (Sigma-Aldrich) for 5 min. Non-specific proteins were then blocked with 10% normal serum with 0.1% Triton X-100 at room temperature for 1 h and stained with the primary antibodies against human DDX11 (Sigma-Aldrich) or PARP (Cell Signaling Technology) that could detect total full-length PARP-1. After washing, slides were incubated with Alexa Fluor 555- or fluorescein-labeled secondary antibodies (Vector Laboratories, Burlingame, CA, USA) and mounted with VECTASHIELD^®^ Mounting Medium (Vector Laboratories). The immunofluorescence images were captured using a Zeiss LSM700 confocal microscope (Carl Zeiss Microscopy GmbH, Jena, Germany) [12].

### 2.3. DDX11 Small Interfering (si) RNA Transfection

RCC cells were transfected with 5 nM of a custom-synthesized Cy5-conjugated *DDX11* siRNA (Dharmacon, Lafayette, CO, USA) for 24 h and fixed with 4% paraformaldehyde, counterstained with DAPI (Invitrogen, Thermo Fisher Scientific, Waltham, MA, USA), and imaged. The Cy5-conjugated *DDX11* siRNA was designed to target the 5′-CCU GUG UCU GUC UUC CUG CGA A-3′ sequence—a 25 bp region determined using the Basic Local Alignment Search Tool [12]—in exon 3 of human *DDX11* located on chromosome 12p11 [15]. It was conjugated to the fluorophore Cy5 via a Label IT siRNA Tracker Intracellular Localization Kit (Mirus Bio, Madison, WI, USA). Dual color images were processed using Zeiss LSM700 (Carl Zeiss Microscopy GmbH). RCC cells were transfected with the single *DDX11* siRNA (Dharmacon) or a control ON-TARGETplus Non-targeting siRNA Pool (Dharmacon) using Lipofectamine RNAiMAX Transfection Reagent (Invitrogen). In order to rule out possible off-target effects, SMARTpool ON-TARGETplus *DDX11* siRNA (Dharmacon) and Silencer Select *DDX11* siRNA (Thermo Fisher Scientific) were used. The results were the same for all 3 different *DDX11*-targeting siRNAs (Supplementary Figure S1).

### 2.4. qRT-PCR

Total RNA was prepared from DDX11- and control siRNA-transfected RCC cells using the RNeasy Mini Kit (Qiagen, Valencia, CA, USA). The RNase-Free DNase Set (Qiagen) was used to remove genomic DNA contamination from the prepared RNA. Complementary DNA (cDNA) was synthesized using Maxime RT PreMix (iNtRON Biotechnology, Korea) from 1 μg of RNA. For qPCR, primers spanning the human *DDX11* exon boundary 20–22 (Bioneer Corporation, Daejeon, Korea) that generated an 88 bp amplicon were used. Human glyceraldehyde 3-phosphate dehydrogenase primers served as the internal control, and primers against the same were synthesized by Bioneer Corporation. qPCR was performed on a StepOnePlus Real-Time PCR system (Applied Biosystems, Foster City, CA, USA) using Power SYBR Green Master Mix (Thermo Fisher Scientific), and involved 40 qPCR cycles (15 s at 95 °C, 30 s at 58 °C, 1 min at 72 °C); data were analyzed using the 2^−ΔΔCt^ method.

### 2.5. Cell Proliferation and Apoptosis Analysis

RCC cell proliferation was determined using the Cell Counting Kit-8 (CCK-8; Dojindo Laboratories, Kumamoto, Japan) according to the manufacturer’s instructions. Briefly, cells were seeded in 96-well plates at a density of 2000–5000 cells per well and transiently transfected. *DDX11*- as well as control siRNA-transfected cells and cells that had received only Lipofectamine RNAiMAX (Invitrogen) were analyzed in triplicate at 24, 48, and 72 h. Furthermore, *DDX11*- as well as control siRNA-transfected cells were treated with the desired concentrations of the inhibitors (5 µM sunitinib and 10 µM olaparib (Selleckchem, Houston, TX, USA)). To properly examine the effects of PARP inhibitors, sensitivity assays at different drug concentrations were performed [16]. Because cell viability was significantly different in concentrations higher than 10 µM olaparib (Supplementary Figure S2A), we decided to use the concentration of 10 µM. For veliparib, the concentration of 100 µM was used because cell viability was significantly different in concentrations higher than 100 µM veliparib (Supplementary Figure S2B). Each plate was incubated for 24, 48, or 72 h, as indicated. At the end of the experiment, 10 μL of CCK-8 solution was added and incubated for 4 h at 37 °C. Absorbance was detected at 450 nm using a VersaMax Microplate Reader (Molecular Devices, Sunnyvale, CA, USA). For the detection of apoptosis using immunofluorescence, *DDX11*- as well as control siRNA-transfected RCC cells were fixed, permeabilized, labeled using the In Situ Cell Death Detection Kit, TMR red (Roche Applied Science, Indianapolis, IN, USA), counterstained with fluorescent DAPI, and imaged.

## 3. Results

### 3.1. DDX11 Expression in Normal Kidney Tissues, Benign Renal Tumors, and RCC Tissues and Cell Lines

**Figure 1:**
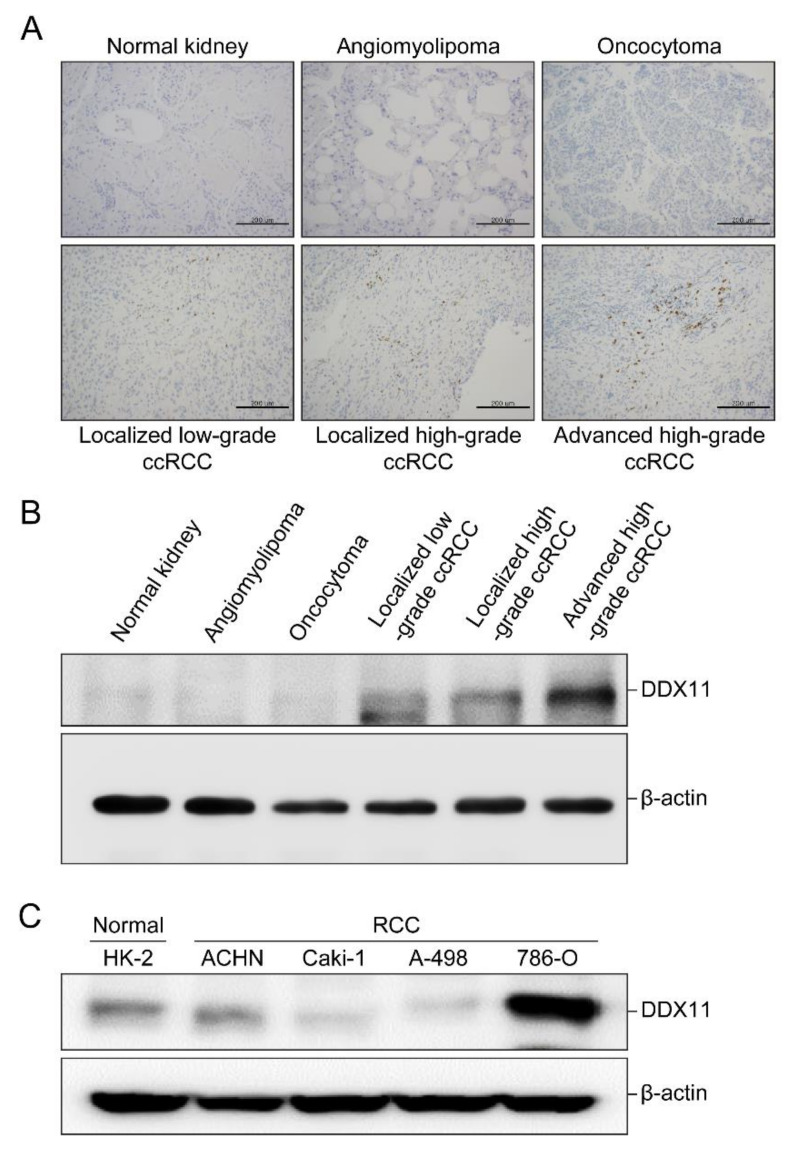
DDX11 expression in normal kidney tissues, benign renal tumors, and renal cell carcinoma (RCC) cell lines and tissues. (**A**) Cryopreserved tissue sections representing normal kidney tissues, benign renal tumors (angiomyolipoma and oncocytoma), localized low-grade clear cell RCC (ccRCC), localized high-grade ccRCC, and advanced high-grade ccRCC were stained with an antibody against human DDX11 and counterstained with hematoxylin. Images were captured at 200 × magnification, Scale bar: 200 µm. (**B**) Immunoblot representing the expression of DDX11 in normal kidney tissues, benign renal tumors, and ccRCC (localized low- and high-grade ccRCC and advanced high-grade ccRCC) tissues. (**C**) Immunoblot representing the expression of DDX11 in RCC cells (786-O, Caki-1, A-498, and ACHN) and the human proximal tubular cell line (HK-2). β-actin was used as the internal control. Original Western Blots can be found in Figure S5.

Immunohistochemistry analysis of cryopreserved tissue sections prepared from normal kidney, benign renal tumors (angiomyolipoma and oncocytoma), localized low-grade ccRCC, localized high-grade ccRCC, and advanced high-grade ccRCC using an antibody against human DDX11 revealed strong expression of DDX11 in advanced and high-grade ccRCC, whereas weak DDX11 expression was noted in localized and low-grade ccRCC (Figure 1A). In contrast, DDX11 expression was not detected in normal kidney tissues or benign renal tumors (Figure 1A).

Subsequent immunoblot analysis revealed strong DDX11 expression in advanced and high-grade ccRCC, but no DDX11 expression was observed in normal kidney tissues or benign renal tumors (Figure 1B).

Immunoblotting revealed that the expression of DDX11 was higher in 786-O cells (RCC) than in other RCC cell lines or HK-2 cells (human proximal tubular cells) (Figure 1C).

**Figure 2:**
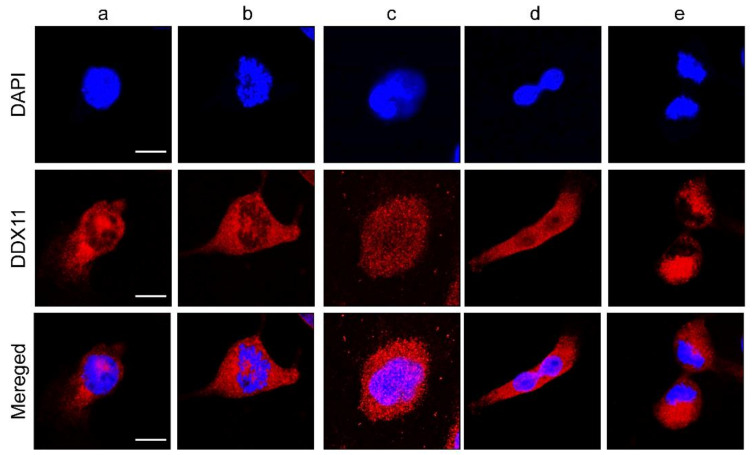
Immunofluorescence analysis representing DDX11 expression in renal cell carcinoma cells. DDX11 expression in 786-O cells during interphase (**a**), prophase (**b**), metaphase (**c**), telophase (**d**), and late telophase (**e**); DDX11 (pseudocolored red), nuclei (pseudocolored blue; DAPI), Scale bar: 10 µm.

Using 786-O cells, which exhibited clearly detectable expression of the 109-kD DDX11 protein, we performed additional DDX11-based immunofluorescence analysis, as DDX11 has been reported to be dynamically localized during mitosis [11]. With no pretreatment for enriching cells in mitosis, DDX11 antibody staining of 786-O cells demonstrated the expression and localization of DDX11 during interphase (Figure 2, lane a) and the subsequent stages of mitosis (Figure 2, lanes b–e).

### 3.2. Downregulation of DDX11 Severely Alters the Morphology and Disrupts the Segregation of RCC Cells

**Figure 3:**
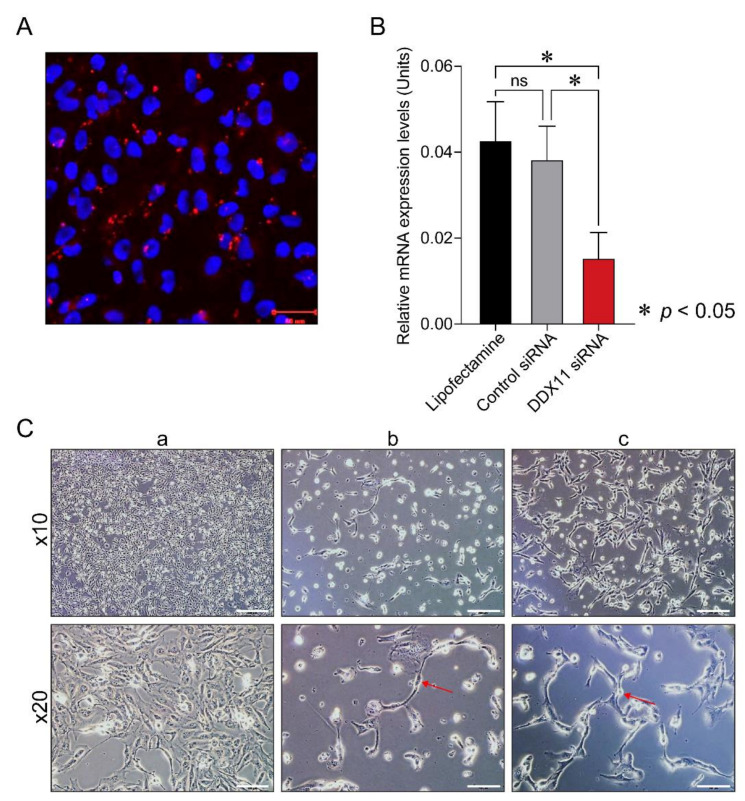
Analysis of *DDX11* small interfering (si) RNA-transfected renal cell carcinoma cells. (**A**) Immunofluorescence to investigate DDX11 expression in 786-O cells 24 h following transfection with 5 nM of Cy5-conjugated *DDX11* siRNA; DDX11 (pseudocolored red), nuclei (pseudocolored blue), Scale bar: 50 µm. (**B**) Quantitative PCR investigating the expression of DDX11 in 786-O cells that received only Lipofectamine (black-colored filled bar) or were transfected with 25 nM of control siRNAs (gray-colored filled bar) or *DDX11* siRNA (red-colored filled bar) for 48 h (* denotes *p* value less than 0.05). (**C**) Phase-contrast images, captured at 20× magnification, depicting the morphology of 786-O cells following transfection with 50 nM of control siRNAs for 96 h (**a**) or with 25 nM (**b**) or 50 nM (**c**) of *DDX11* siRNA for 24 h. The red-colored arrow in the phase-contrast images, shown in panels **b** and **c**, points to the chain-like morphology of 786-O cells that did not separate, Scale bar: 200 µm (upper row), 100 µm (lower row).

We transfected 786-O cells with a previously reported *DDX11*-specific siRNA [12,15]. Imaging was performed 24 h after transfection to ascertain the effective uptake of the *DDX11*-specific siRNA, and the results revealed the presence of *DDX11*-specific siRNA in RCC cells (Figure 3A).

The levels of DDX11 decreased following the transfection of 786-O RCC cells with *DDX11* siRNA (25 nM) compared to those in the control (786-O cells transfected with 25 nM of a pool of siRNAs comprising four non-targeting siRNAs). qPCR analysis 48 h after transfection revealed that the expression of DDX11 decreased in 786-O cells transfected with *DDX11*-specific siRNA compared to that in 786-O cells that had only received Lipofectamine RNAiMAX or the control siRNA pool (Figure 3B; *p* = 0.0091).

RCC cells transfected with the *DDX11* siRNA exhibited a rapid and dramatic alteration in their morphology compared to the control siRNA-transfected cells (Figure 3C, panel a). In particular, we found that *DDX11* siRNA transfection caused RCC cells to lose cell-cell contact in as little as 24 h and at doses of 25 nM and 50 nM. Moreover, a significant number of transfected RCC cells exhibited a tightly arranged chain-like morphology (Figure 3C, panels b and c).

### 3.3. DDX11 Knockdown Severely Inhibits RCC Cell Proliferation and Induces Apoptosis of RCC Cells

**Figure 4:**
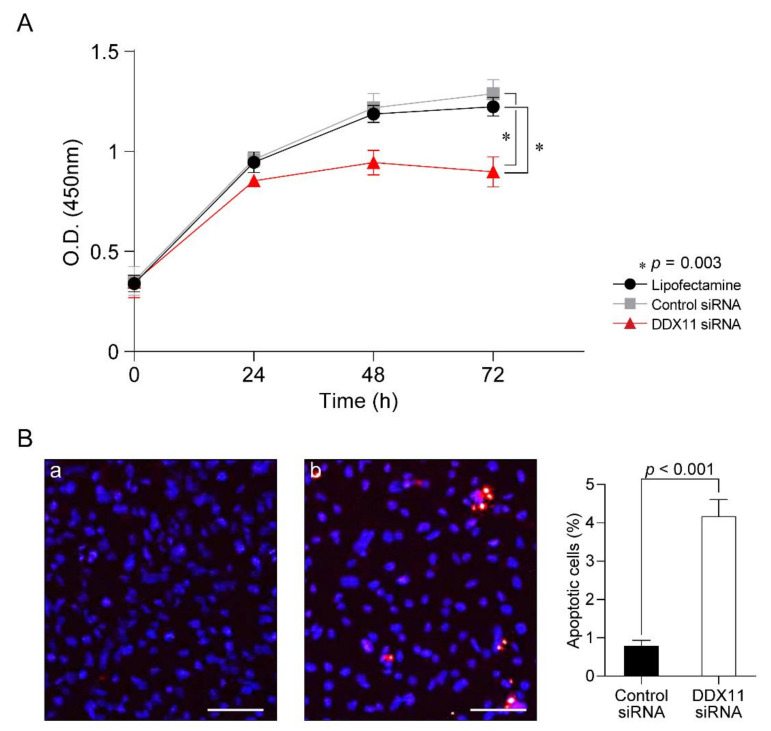
*DDX11* knockdown in renal cell carcinoma cells resulted in the inhibition of proliferation and apoptosis. (**A**) Proliferation of 786-O cells that received only Lipofectamine (black-colored line) or were transfected with 25 nM of control siRNAs (gray-colored line) or 25 nM of *DDX11* siRNA (red-colored line). The value at each time point following siRNA transfection is the mean of triplicate samples analyzed (* denotes *p* value with 0.003). (**B**) Apoptosis of 786-O cells transfected for 48 h with 50 nM of control siRNAs (**a**) or 50 nM of *DDX11* siRNA (**b**) was analyzed using the TUNEL assay; TUNEL-positive cells (pseudocolored red), nuclei (pseudocolored blue), scale bar: 100 µm. Apoptosis of 786-O cells transfected for 48 h with 50 nM of control siRNAs (**a**) or 50 nM of *DDX11* siRNA (**b**) was analyzed using the TUNEL assay.

We transfected RCC cells (5000 cells/96-well plate) with 25 nM each of *DDX11* or the pool of control siRNAs; the rates of cell proliferation were measured 24 h after transfection and every 24 h thereafter. As shown in Figure 4A, compared to RCC cells that had received only Lipofectamine RNAiMAX or were transfected with the control siRNAs, RCC cells transfected with *DDX11*-specific siRNA exhibited strongly inhibited proliferation even up to 72 h post-transfection (*p* = 0.003).

The most severe *DDX11* siRNA-induced change was that the cells rapidly and massively underwent apoptosis, as demonstrated by the terminal deoxynucleotidyl transferase-mediated dUTP nick-end labeling (TUNEL) assay. RCC cells that had been transfected for 48 h with 50 nM of the pooled siRNA did not reveal any apoptosis, but RCC cells transfected with *DDX11* siRNA clearly exhibited apoptosis with significance (*p* < 0.001) (Figure 4B).

### 3.4. DDX11 Knockdown in RCC Cells Induces Sensitivity to PARP Inhibition

**Figure 5:**
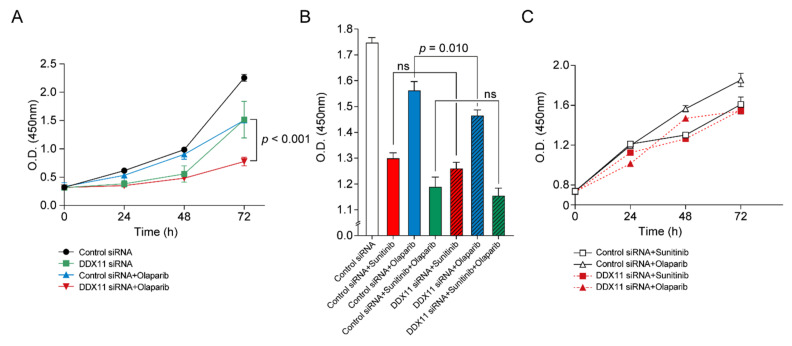
*DDX11* knockdown in renal cell carcinoma cells induced sensitivity to poly (ADP-ribose) polymerase (PARP) inhibitors. (**A**) Proliferation curves of 786-O cells transfected with the control siRNAs or *DDX11* siRNA following olaparib treatment after 24, 48, and 72 h. (**B**) Cell viability after exposure to sunitinib, olaparib, or a combination of both drugs in the background of transfection with *DDX11* siRNA or control siRNA for 48 h (ns, not significant). (**C**) Proliferation curves of 786-O cells transfected with the control siRNAs or with the *DDX11* siRNA after 24, 48, and 72 h of sunitinib or olaparib treatment.

RCC cells transfected with *DDX11* siRNA or control siRNA were treated with a clinically approved PARP inhibitor, olaparib; subsequently, cell proliferation was evaluated to investigate the association between DDX11 expression and the sensitivity to PARP inhibition. In response to treatment with olaparib alone, *DDX11* siRNA-transfected RCC cells exhibited decreased viability (starting from 48 h) compared to those transfected with the control siRNAs and mostly showed significant differences (*p* < 0.001; 72 h time point). Thus, RCC cells with *DDX11* knockdown exhibited increased sensitivity to olaparib (Figure 5A).

Furthermore, viability was evaluated upon exposure of RCC cells to sunitinib, olaparib, or both drugs in combination with *DDX11* siRNA transfection or with control siRNA transfection. As shown in Figure 5B, a significant difference (*p* = 0.010, 48 h time point) in cell viability was observed following olaparib treatment; however, no differences were observed when the cells were treated with sunitinib alone or with a combination of sunitinib and olaparib. Proliferation curves revealed that the efficacy of olaparib was similar to that of sunitinib in RCC cells transfected with *DDX11* siRNA. Furthermore, when *DDX11* was knocked down, olaparib exerted effects similar to those of sunitinib (Figure 5C). For comparison of the efficacy of other PARP inhibitors on *DDX11* siRNA transfected cells, veliparib was used. Veliparib demonstrated efficacy similar to olaparib in *DDX11* knockdown RCC cells (*p* < 0.001; 72 h time point, Supplementary Figure S3A). Moreover, a significant difference (*p* = 0.019, 48 h time point) in cell viability was only observed between groups with *DDX11* siRNA transfection or with control siRNA transfection following veliparib treatment (Supplementary Figure S3B).

### 3.5. DDX11 Knockdown in RCC Cells Increases PARP Protein Expressions in Nucleus

RCC cells transfected with *DDX11* siRNA or control siRNA were treated with a clinically approved PARP inhibitor, olaparib; subsequently, immunofluorescence staining of PARP was performed to measure and investigate the increased PARP expression in nuclei of *DDX11* knockdown RCC cells. PARP expression was increased in *DDX11* siRNA-transfected RCC cells compared to control siRNA-transfected cells. In response to treatment with olaparib, PARP expression was decreased both in RCC cells with *DDX11* siRNA transfection or with control siRNA transfection (Supplementary Figure S4).

## 4. Discussion

This is the first study to demonstrate that the expression of the DEAD/DEAH box helicase, DDX11, is upregulated during progression from early to advanced ccRCC. Our findings revealed the important role played by DDX11 in preventing chromosomal segregation and apoptosis in advanced ccRCC cells, thereby maintaining the aggressiveness of this cancer. Furthermore, the novel and important findings described herein reveal that DDX11 is the determinant of PARP inhibitor sensitivity in RCC.

We focused on identifying biomarkers associated with the progression of localized low-grade ccRCC to advanced high-grade ccRCC. We identified 10 genes in samples isolated from archived, formalin-fixed, and paraffin-embedded tissue samples representing aggressive ccRCC with synchronous metastasis, recurrence, or cancer-specific death versus those with non-aggressive ccRCC [9]. Our analysis revealed that the expression of *DDX11*—a gene not known to be associated with ccRCC—was upregulated in aggressive ccRCC compared to that in non-aggressive ccRCC, and that this upregulation was significantly related to poor overall survival (*p* < 0.05) [9]. Furthermore, a prospective validation cohort study (NCT03694912) involving 140 patients revealed that the levels of *DDX11* mRNA in tissues as well as in the plasma are significantly associated with high-grade ccRCC [10].

*DDX11* (ChlR1), first isolated as the human homolog of the yeast *CHL1* gene, is a member of the DEAD/DEAH box family of helicases. It is required for the cohesion of chromosomal arms and centromeres, thereby playing an important role in maintaining genome stability [11,17,18,19]. The Pisani group and collaborators demonstrated that DDX11 functionally interacts with Timeless, a subunit of the replication-fork protection complex, to preserve fork integrity [20], and recent study reported that DDX11 resolves problems occurring at the replication forks [21]. Biochemical characterization of DDX11, produced in recombinant form, revealed that it has an ATPase-dependent DNA unwinding activity with a 5′ to 3′ directionality in vitro and is also able to dismantle unconventional DNA structures [22,23,24,25,26,27]. However, to date, little is known regarding the role of helicases in ccRCCs. DDX11 loss has been reported to result in alterations in telomeric chromatin formation [19]. Furthermore, DDX11 interacts with the flap structure-specific endonuclease 1 (FEN-1) [24], an event that is pivotal to ensure telomere stability. Thus, similarly to DDX39—which is associated with telomere lengthening [28]—DDX11 plays an important role in maintaining telomere length and stability in malignancies such as melanoma, lung cancer, and RCC [12,13]. Bhattacharya et al. [12] reported that high DDX11 expression was significantly associated with poor prognosis in advanced melanomas. Moreover, similarly to the findings observed in this study, Li et al. [13] reported that DDX11 was significantly upregulated and predicted poor prognosis in lung adenocarcinoma. Besides melanoma and lung cancer, associations with hepatocellular carcinoma (HCC) and osteosarcoma have been reported [22]. Two recent studies have reported the association of DDX11 in HCC development and progression [29,30]. Yu et al. suggested a pro-tumorigenic role of DDX11 in xenograft cancer animal models and demonstrated that DDX11 overexpression leads to activation of PI3K/AKT/mTOR signaling pathway [29].

Here, we have shown that the inhibition of DDX11 results in rapid RCC cell apoptosis. Although this finding has been reported in studies on DDX11^-/-^ mice, which show that DDX11 depletion induces apoptosis [31,32], no other studies—except those on melanoma [12]—have shown that rapid programmed cell death is induced in response to DDX11 downregulation.

Numerous efforts have been made to improve the survival of patients with metastatic RCC (mRCC), and enhanced survival has been clearly documented in this era of targeted therapies and immunotherapies [4,6]. The currently available mRCC-targeted therapies include vascular endothelial growth factor (VEGF) tyrosine kinase inhibitors, anti-VEGF monoclonal antibodies, and mammalian target of rapamycin inhibitors [4,6]. Contrary to traditional immunotherapies, such as interferon-α and interleukin 2, which are associated with excessive toxicity, modern immunotherapies, such as antibodies against programmed cell death protein 1, programmed death-ligand 1, or cytotoxic T-lymphocyte-associated protein 4, disable the ability of tumor cells to evade the immune system without any significant side effects [33,34,35]. Currently, 15 different therapeutic approaches, 10 agents for first-line treatment, and 11 agents for further-line treatment have been approved for mRCC [36].

However, despite these improvements and increased therapeutic options, the selection of treatments for individual patients is becoming more challenging. This is because of an apparent paradox in modern oncology: the targeted treatments are employed without assessing the genomic profile of the tumors in individual patients [4,6]. Several studies have identified and incorporated gene expression signatures and mutation status into the decision-making protocol regarding treatment [7,37,38]. *PBRM1* mutation is associated with favorable responses to antiangiogenic therapies [37,38], while *BAP1* mutation is associated with poor response to antiangiogenics due to reduced angiogenic signaling [37]. However, at present, molecular profiling is not performed in routine clinical settings to enable personalized treatment [6]. Therefore, we focused on identifying clinically useful biomarkers that would guide the selection of treatment options.

“BRCAness” is a term used to describe tumors with a defect in DNA double-strand break repair by homologous recombination, which mimic defects in *BRCA* [39,40,41]. The positive response of *BRCA*-mutated breast and ovarian cancers to PARP inhibitors [42,43] has raised interest in identifying additional determinants of PARP inhibitor sensitivity, thereby extending the utility of PARP inhibitors in cancer therapy [39]. However, the use of PARP inhibitors has not been approved for the treatment of mRCC, and only a few clinical trials (e.g., NCT03786796) are investigating the effects of the PARP inhibitors on mRCC. Only one in vitro study has suggested the possible use of PARP inhibitors for RCCs [39]. Scanlon et al. [39] demonstrated that von Hippel–Lindau (VHL) deficiency—present in 60–80% of ccRCC patients—is associated with increased sensitivity to PARP inhibitors.

Similarly, our study demonstrated that decreased DDX11 expression in RCC increases the sensitivity to PARP inhibitors, indicating that *DDX11*-deficient RCCs share some features with BRCAness tumors. Furthermore, our study is the first to report the increased PARP expression in nuclei of *DDX11*-deficient RCC cells, demonstrating that DDX11 downregulation renders RCCs sensitive to PARP inhibitor and that DDX11 is involved in the repair of PARP trapping. *DDX11*-depleted RCC cells were more vulnerable to olaparib than to sunitinib alone or to a combination of sunitinib plus olaparib. Moreover, we have reported that olaparib and sunitinib exhibited similar efficacies against *DDX11*-deficient RCC cells; this result is novel because PARP inhibitors have not been recommended for the treatment of RCC to date. Therefore, investigations of PARP inhibitors in *DDX11*-deficient RCC animal models must be performed to enable future clinical applications.

A possible mechanism that could explain the increased PARP inhibitor sensitivity in *DDX11*-deficient RCC cells is the involvement of DDX11 in sister chromatid cohesion during replication in the S phase [44]. The replication fork protection complex stabilizes DDX11, leading to the stable association of the cohesion complex with chromatin [15]. Moreover, DDX11 enhances the activity of FEN1, which is involved in lagging-strand DNA synthesis [24]. As the depletion of FEN1 results in the development of cohesion defects, and FEN1-deficient cells are sensitive to PARP inhibitors [24,45], we inferred that lagging-strand synthesis might be crucial for sister chromatid cohesion. In summary, DDX11 deficiency results in the development of defects in sister chromatid cohesion and lagging-strand DNA synthesis, thereby comprehensively increasing PARP inhibitor sensitivity.

We used the 786-O cell line for our analysis because it harbors a mutated *VHL* [46,47] with altered hypoxia-inducible factor 1 and VEGF pathways. As demonstrated in a previous study, *VHL*-deficient renal cells and *VHL*-deficient RCC tissue samples exhibit reduced expression of homologous recombination- and mismatch repair-related genes, and increased sensitivity to PARP inhibitor [39]. Our study showed that *VHL*-deficient 786-O cells became more susceptible to the PARP inhibitor olaparib in the background of DDX11 downregulation.

Because DDX11 is a nuclear protein, our results showed clear localization of DDX11 in the nucleus, especially during interphase, prophase, and metaphase. However, localization of DDX11 in the cytoplasm was also noted. This might be due to the dynamic localization of DDX11 during mitosis [11,12]. Parish et al. [11] and Bhattacharya et al. [12] reported dynamic localization of DDX11 during mitosis which is similar to our study. Further investigations on why nuclear protein, DDX11, is becoming localized in cytoplasm during mitosis should be performed.

There were a few limitations in this study. First, we could not clinically validate the efficacy of olaparib in *DDX11*-deficient RCC patients. It would be more promising if olaparib had demonstrated its superior efficacy in DDX11-overexpressed ccRCC compared to DDX11-downexpressed ccRCC, because the use of olaparib would not be limited to non-aggressive ccRCC patients, who have fewer options for targeted therapy compared to those with aggressive ccRCC. However, no other studies have suggested the use of PARP inhibitor in ccRCC. Therefore, this study has its significance in suggesting the use of PARP inhibitor as an option for targeted therapy in ccRCC patients. Future clinical studies of *DDX11*-deficient RCC patients who are refractory to conventional treatments and can be treated with olaparib are planned to address this issue. Second, owing to limited funding and time, we could not develop a *DDX11*-deficient xenograft model using CRISPR/Cas9. Third, BRCA 1 and 2 could not be evaluated in this study due to the technical difficulty and lack of previous studies reporting an association of BRCA 1 and 2 with RCC [40,41]. Therefore, the evaluation and knockdown of *BRCA* 1 and 2 ranks as an independent theme for large studies to be performed in the future. Fourth, we demonstrated that PARP expression was increased in the nucleus after *DDX11* knockdown, despite the unknown association with chromatin. Demonstration of PARP retention on chromatin would be better presented with the method used in the study by Muari et al. [45]; that approach will be incorporated in a future study. Despite its weaknesses, our study is the first to demonstrate the role of DDX11 in ccRCC and the use of PARP inhibitors for treatment of *DDX11*-deficient ccRCC.

## 5. Conclusions

To the best of our knowledge, this is the first study to demonstrate that DDX11 is expressed at high levels in advanced RCC, but not in normal kidney tissues or benign renal tumors. Furthermore, we found that the downregulation of DDX11 induced segregation defects, inhibition of cell proliferation, and rapid RCC cell apoptosis. Finally, we identified determinants of PARP inhibitor sensitivity in RCC, which have not been previously reported. Collectively, these results suggest that *DDX11* represents a new therapeutic biomarker for the treatment of mRCC that is refractory to conventional targeted therapies and immunotherapies.

## Data Availability

The datasets used and/or analyzed in this study are available from the corresponding author upon reasonable request.

## Supplementary Materials

The following are available online at https://www.mdpi.com/article/10.3390/cancers13112574/s1, Figure S1: Immunoblot representing the expression of DDX11 in RCC cells transfected with 3 different *DDX11*-targeting siRNAs (*DDX11* siRNA #1, custom-synthesized *DDX11* siRNA (Dharmacon); *DDX11* siRNA #2, SMARTpool ON-TARGETplus *DDX11* siRNA (Dharmacon); *DDX11* siRNA #3, Silencer Select *DDX11* siRNA (Thermo Fisher Scientific)). β-actin was used as the internal control, Figure S2: (A) PARP inhibitor sensitivity of 786-O cells that transfected with control siRNAs (black-colored line) or *DDX11* siRNA (red-colored line) treated with increasing concentrations of olaparib. (B) PARP inhibitor sensitivity of 786-O cells that transfected with control siRNAs (black-colored line) or *DDX11* siRNA (red-colored line) treated with increasing concentrations of veliparib, Figure S3: (A) Proliferation curves of 786-O cells transfected with the control siRNAs or *DDX11* siRNA following veliparib treatment after 24, 48, and 72 h. (B) Cell viability after exposure to sunitinib, veliparib, or a combination of both drugs in the background of transfection with *DDX11* siRNA or control siRNA for 48 h, Figure S4: 786-O cells that transfected with control siRNA or *DDX11* siRNA treated with olaparib for 24 h followed by staining against PARP. Representative immunofluorescence staining image of PARP (pseudocolored green) and DAPI (pseudocolored blue), Scale bar: 10 µm. Figure S5: Original Western Blots.
