# Supplementary figures and images for: The DEAD/DEAH Box Helicase, DDX11, Is Essential for the Survival of Advanced Clear Cell Renal Cell Carcinoma and Is a Determinant of PARP Inhibitor Sensitivity
Source: Cancers (Basel). 2021 May 24;13(11):2574. doi: 10.3390/cancers13112574 (PMC8197413; doi:10.3390/cancers13112574)

Figure 1\_B

DDX11

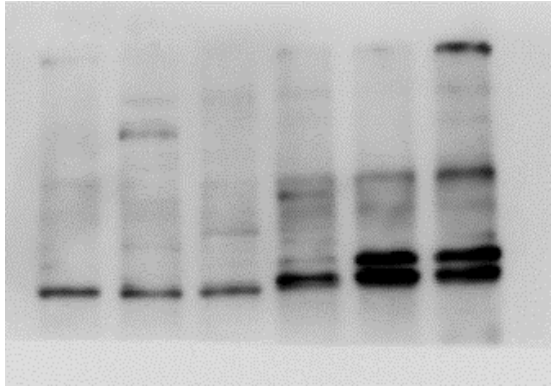

$\beta$ -actin

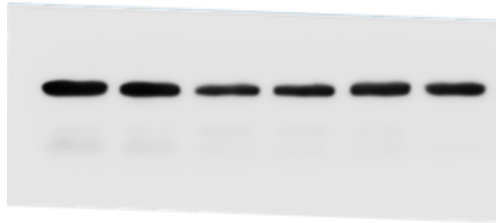

Figure 1\_C

DDX11

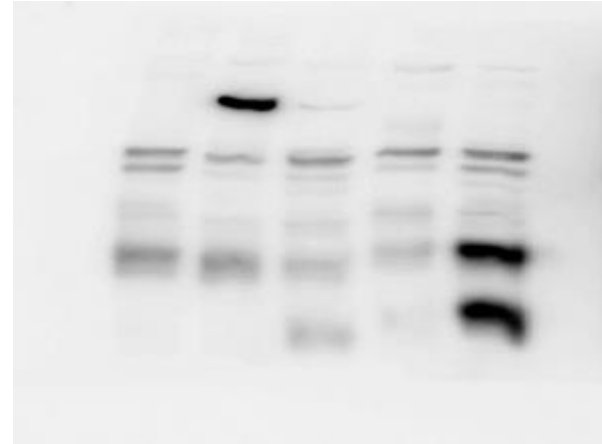

$\beta$ -actin

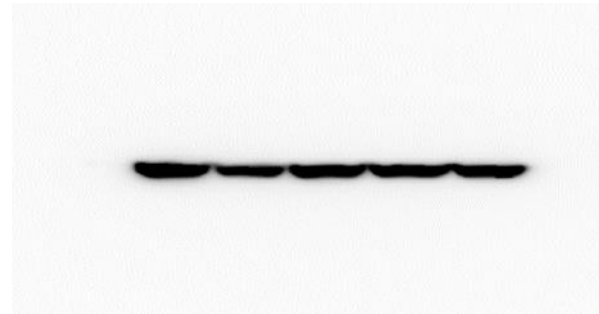

Supplement: Supplementary file 1 [file cancers-13-02574-s001.zip › Figure S5.pdf]

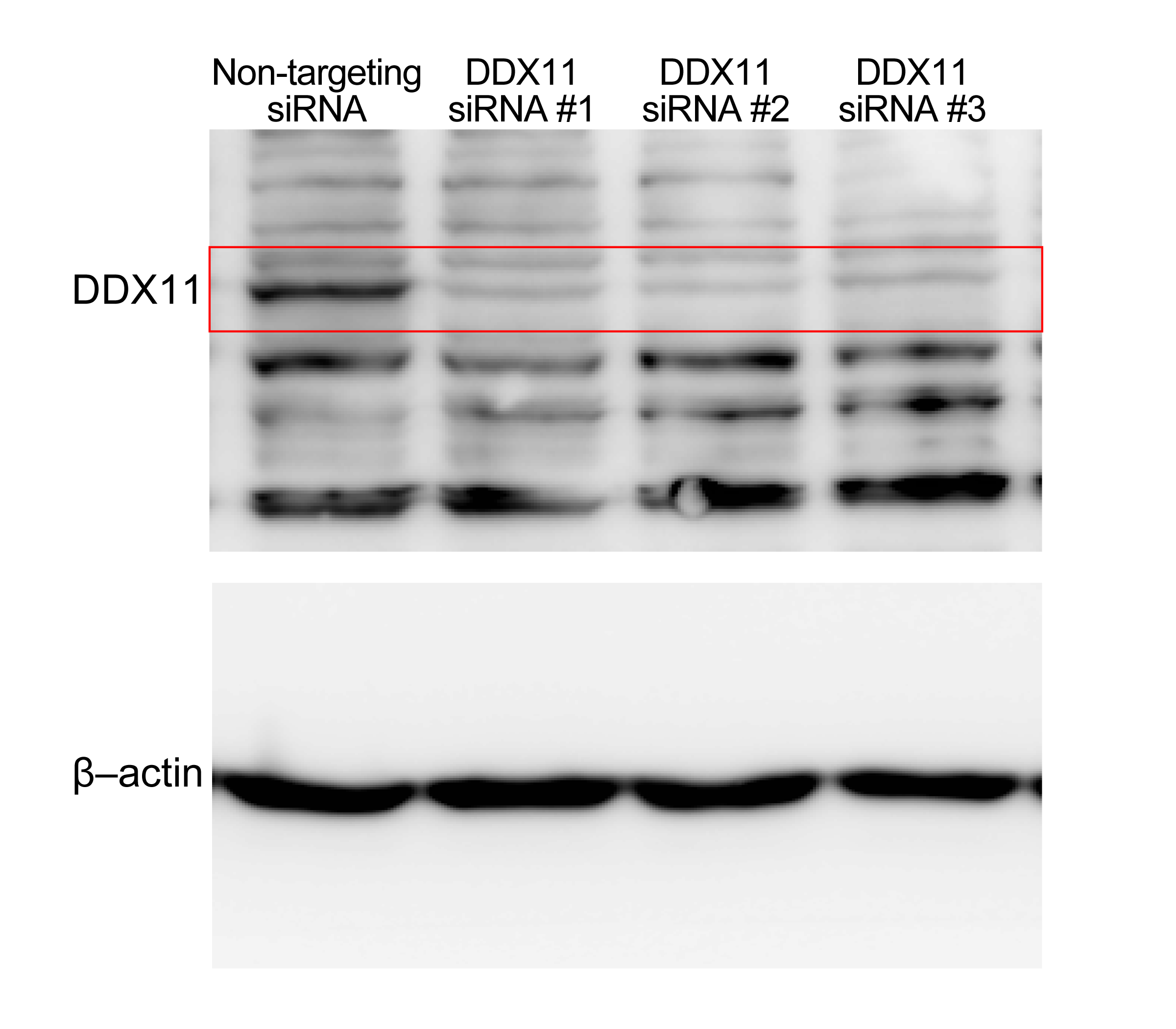

Supplement: Supplementary file 1 [file cancers-13-02574-s001.zip › FigureS1.tif]

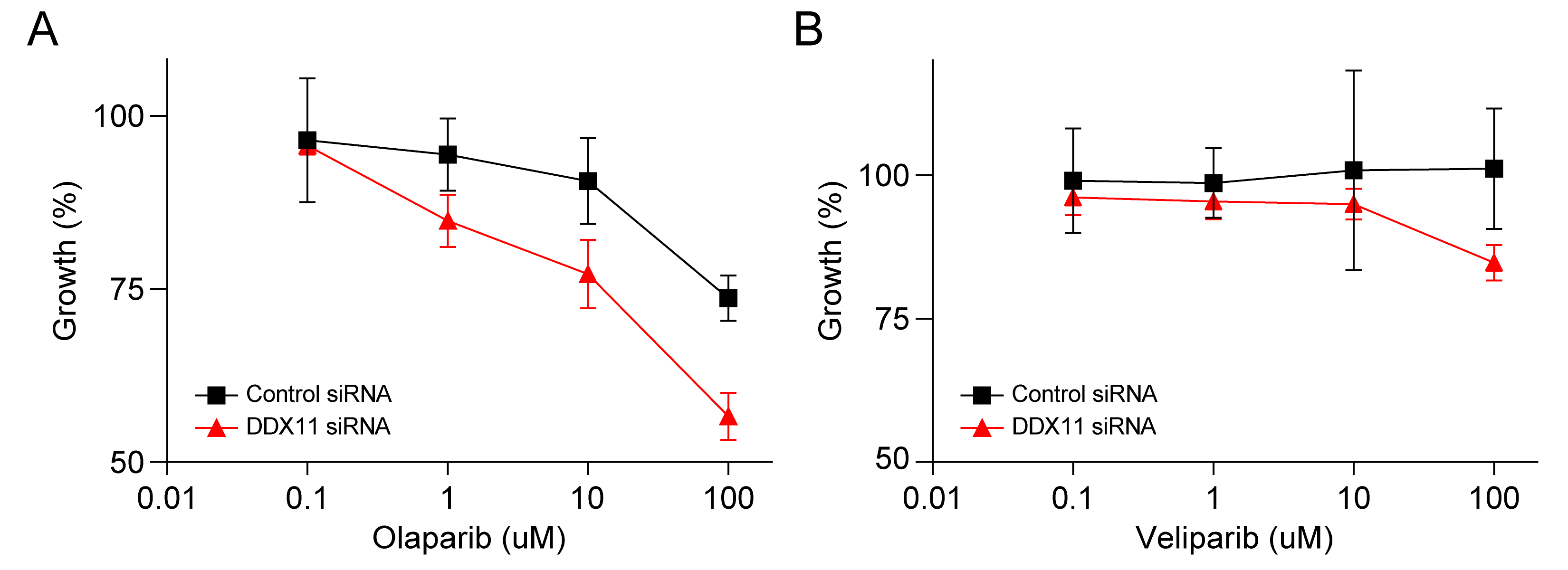

Supplement: Supplementary file 1 [file cancers-13-02574-s001.zip › FigureS2.tif]

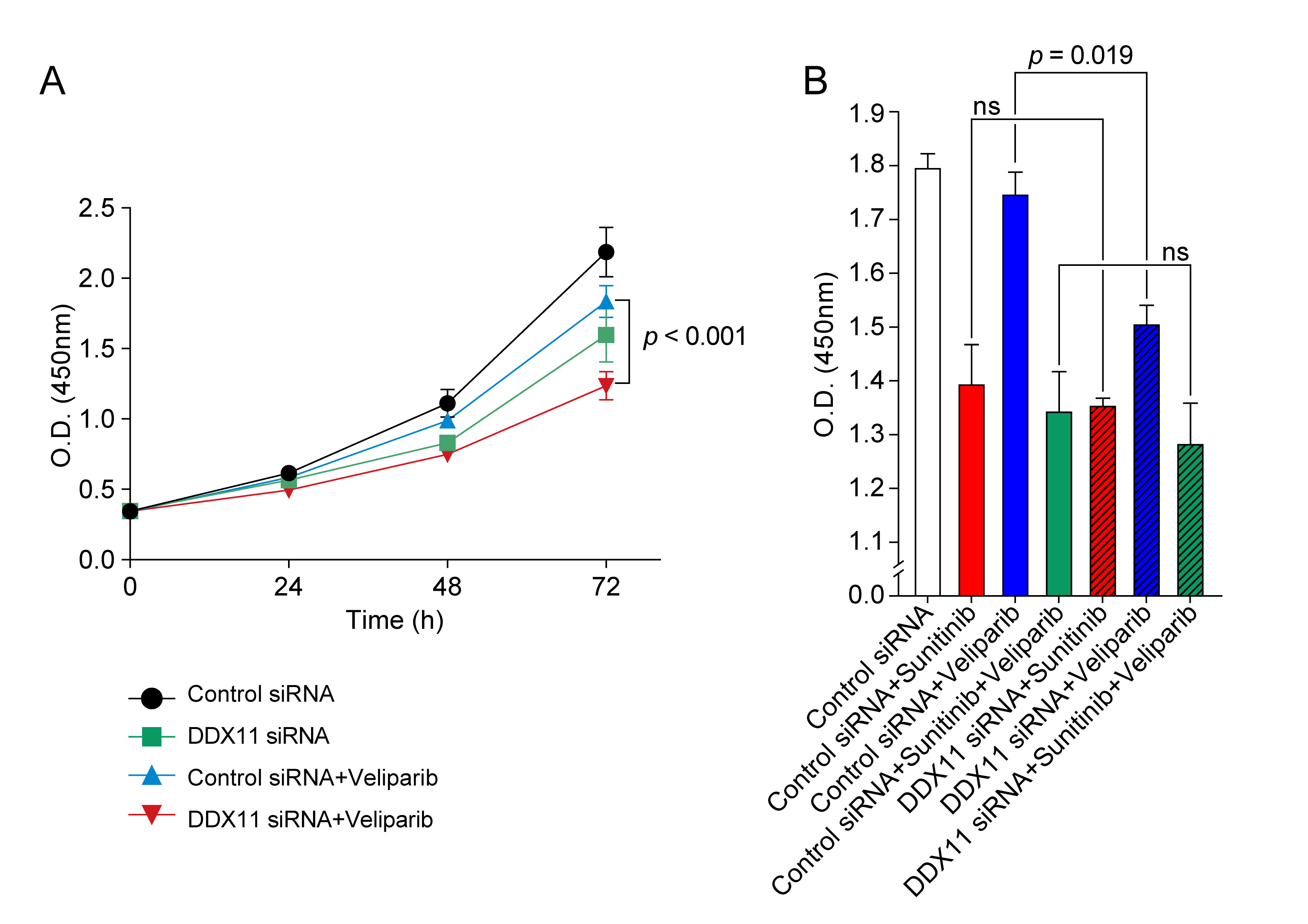

Supplement: Supplementary file 1 [file cancers-13-02574-s001.zip › FigureS3.tif]

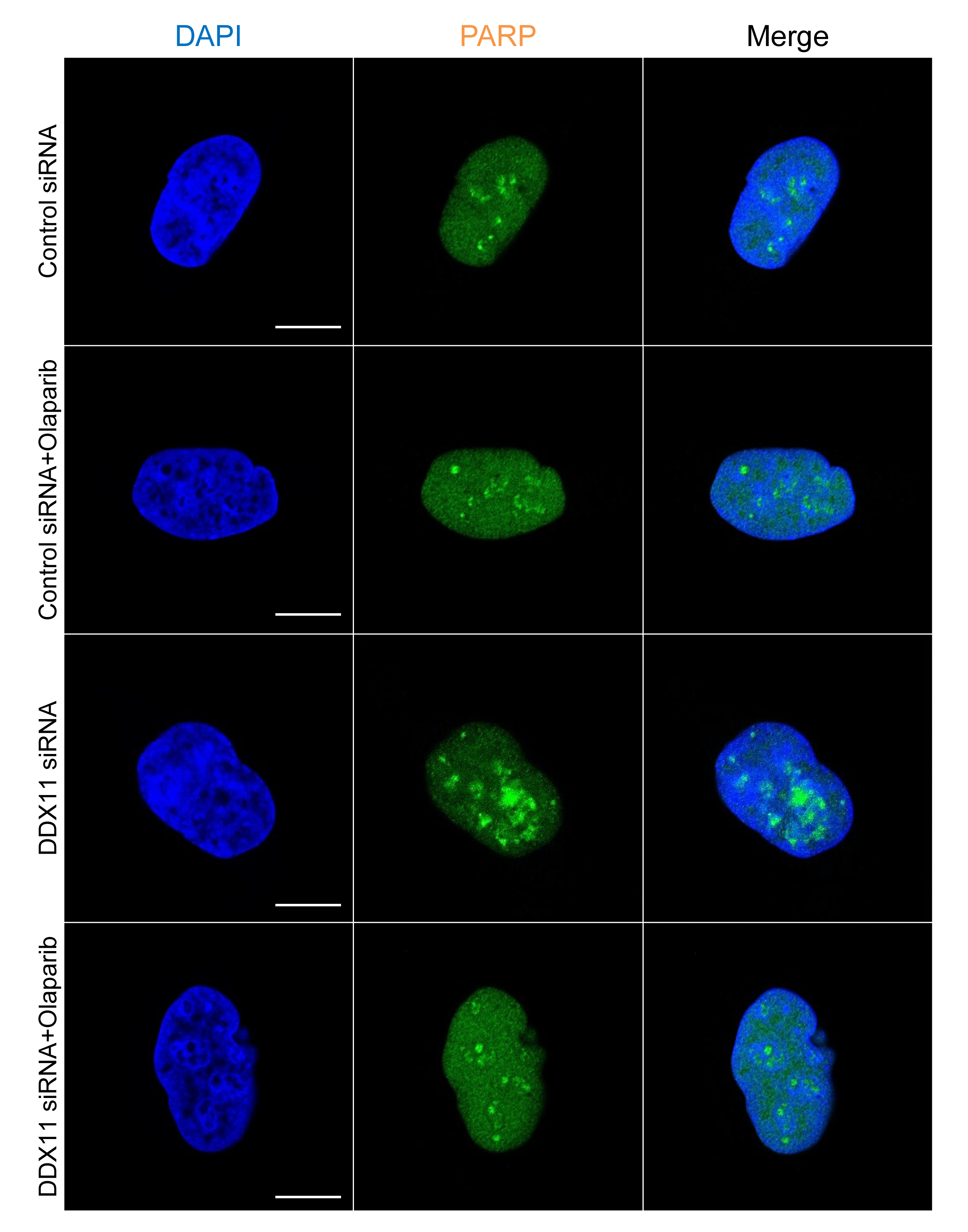

Supplement: Supplementary file 1 [file cancers-13-02574-s001.zip › FigureS4.tif]
